# REFLEX, a social-cognitive group treatment to improve insight in schizophrenia: study protocol of a multi-center RCT

**DOI:** 10.1186/1471-244X-11-161

**Published:** 2011-10-05

**Authors:** GHM Pijnenborg, Mark Van der Gaag, Claudi LH Bockting, Lisette Van der Meer, André Aleman

**Affiliations:** 1 Dept. of Psychotic Disorders, GGZ-Drenthe, Dennenweg 9, 9404 LA, Assen, the Netherlands; 2 Dept. of Clinical Psychology, University of Groningen, Grote Kruisstraat 2/1, 9712 TS, Groningen, the Netherlands; 3 Lentis, Center for Mental Healthcare, Department of Longterm Rehabilitation, Zuidlaren, the Netherlands; 4 Neuroimaging Center, University Medical Center Groningen, P.O. Box 30.001, 9700 RB, Groningen, Groningen, the Netherlands; 5 VU University and EMGO+ Institute of Health and Care Research, Dept. of Clinical Psychology, Van der Boechorststraat 1, 1081 BT Amsterdam, the Netherlands; 6 Parnassia Psychiatric Institute, Prinsegracht 63, 2512 EX The Hague, the Netherlands

**Keywords:** schizophrenia, insight, treatment, self-reflection, self-stigma, perspective-taking

## Abstract

**Background:**

Insight is impaired in a majority of people with schizophrenia. Impaired insight is associated with poorer outcomes of the disorder. Based on existing literature, we developed a model that explains which processes may possibly play a role in impaired insight. This model was the starting point of the development of REFLEX: a brief psychosocial intervention to improve insight in schizophrenia. REFLEX is a 12-sessions group training, consisting of three modules of four sessions each. Modules in this intervention are: "coping with stigma", "you and your personal narrative", and "you in the present".

**Methods/Design:**

REFLEX is currently evaluated in a multicenter randomized controlled trial. Eight mental health institutions in the Netherlands participate in this evaluation. Patients are randomly assigned to either REFLEX or an active control condition, existing of cognitive remediation exercises in a group. In a subgroup of patients, fMRI scans are made before and after training in order to assess potential haemodynamic changes associated with the effects of the training.

**Discussion:**

REFLEX is one of the few interventions aiming specifically to improving insight in schizophrenia and has potential value for improving insight. Targeting insight in schizophrenia is a complex task, that comes with several methodological issues. These issues are addressed in the discussion of this paper.

**Trial registration:**

Current Controlled Trials: ISRCTN50247539

## 1. Background

The percentage of persons with schizophrenia who have only limited insight into their illness is large, ranging from 50-80% [1]. Insight is considered a combination of a number of dimensions, that can fluctuate independently of each other, including awareness of mental illness, relabeling of symptoms and awareness of need for treatment [2]. Insight in schizophrenia is usually measured with a semi-structured interview, such as the SAI-E [3], SUM-D [4], and item G12 of the PANSS-interview [5], or self-rating questionnaires, such as the Beck Cognitive Insight Scale [6], and the Psychosis Insight Scale [7].

Poor insight has a negative impact on relevant outcomes of the disorder [see for a review: [8]]. Poor treatment compliance in patients mediates this relationship, but there is also a direct association between insight and outcome [9]. Limited insight has been associated with more positive and negative symptoms [10], more relapse and rehospitalizations [9], lower GAF-scores [11], and better observer quality of life and social functioning [9]. However, good insight may also have unfavorable consequences. Several studies have shown better insight to be associated with more depressive symptoms [8]. The exact nature of this relationship remains unclear [8,10]. The relationship between depression and insight is thought to be mediated by internalized stigma: insight is only associated with depression in patients who hold stigmatizing beliefs about mental illness [12,13].

Given the negative impact of limited insight on the outcome of schizophrenia, insight is a logical target for treatment. However, treatment options to enhance insight are limited. Psycho-education does not necessarily lead to better insight [14], neither does psycho-dynamic psychotherapy [15]. Turkington et al. [16] developed a treatment program that combines psycho-education on medication with cognitive behavioral therapy. Treatment adherence improved and patients were better able to label their symptoms as psychotic both immediately and one year after treatment. In others studies, no clear effects of cognitive behavioural therapy on insight was found [17].

Kemp et al. [18] demonstrated that therapy adherence and insight in symptoms improved after a brief intervention based on the principles of motivational interviewing. Others studied the same intervention, with inconsistent results [19,20]. Two smaller studies showed that when patients are confronted with video images of themselves during a psychotic episode, their insight improves [21,22]. In sum, although there are several interventions aiming to enhance insight in schizophrenia, there is still a need for improvement.

Three types of models have been put forward to explain this variance: the clinical model, the neuropsychological model, and the psychological denial model [23]. The clinical model suggests that poor insight is a primary symptom of schizophrenia, analogous to delusions and hallucinations. The neuropsychological model argues that specific cognitive impairments are responsible for poor insight in schizophrenia [24,25]. Finally, the psychological denial model explains poor insight as the outcome of a coping strategy that is used to reduce the distress associated with a diagnosis of schizophrenia [26].There is limited support for the clinical model, partly because of the lack of testable hypotheses. Literature does provide evidence for the neuropsychological model and some preliminary support for the psychological denial model [23], but none of these models alone can account for the variance in insight.

Recent evidence [27] suggests that one aspect of cognitive functioning may have been overlooked in insight literature: social cognition. Social cognition refers to "the mental operations underlying social interactions, like the ability and capacity to perceive the intentions and dispositions of others" [28]. In particular, the ability to take perspective has been linked to insight[[29]; Pijnenborg, Spikman, Jeronimus and Aleman: Insight in schizophrenia: the role of affective perspective taking and empathy, submitted]. In other words: the ability to infer mental states was associated with the tendency to take another person's perspective on oneself.

**Figure 1:**
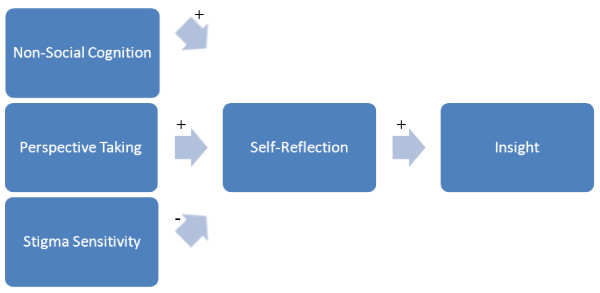
**A model of impaired insight in schizophrenia**.

Based on these findings, we propose a model that integrates elements from previous models and combines them with recent findings on the role of social cognition in insight [30]. According to this model (see Figure 1) self-reflection moderates the relationship between the prerequisites for insight on the one hand and insight on the other. Self-reflection is considered a meta-cognitive process that concerns the ability to reflect upon thoughts and feelings [31]. Self-reflection is thought to be impaired in schizophrenia; patients demonstrate difficulties in generating personal narratives that link the past with the present [32,33]. The model explains why schizophrenia patients with poor insight erroneously hold on to their pre-morbid self-image. Because these patients do not adjust their self-images to changing circumstances, they implicitly assume that functioning and future perspective are still the same as before their illness started. In other words: they make too few self-corrections. A number of processes are thought to hamper self-reflection in schizophrenia. First of all, poor insight is associated with a lack of mental flexibility [25].We propose that this relationship is mediated by self-reflection. A lack of mental flexibility will hamper the capacity to consider alternatives and make complex inferences about oneself, which will inevitably result in poor insight. Second, recent evidence shows that insight in schizophrenia is associated with Theory of Mind (ToM) and in particular the ability to take the perspective of others [34]. ToM refers to the ability to interpret mental states of others, or the notion that mental representations of the world do not necessarily reflect reality, and can be different from one's own [35]. According to David [36], insight requires a capacity for self-reflection and the ability to make self-evaluations. David quotes 18^th ^century Scottish poet Robert Burns to illustrate that the ability to '*see oursels as others see us' *helps people in making these evaluations about themselves. 'Seeing yourself through the eyes of others' is a process that overlaps with ToM, and in particular with the ability to take the perspective of another person to evaluate your own mental state. Indeed, schizophrenia patients are found to recognize symptoms of mental illness in others, but not in themselves [37]. However, a direct link between insight and perspective-taking is thought to be unlikely, as perspective-taking is not primarily intended for self-evaluation [29]. In line with our model, Langdon and Ward suggest self-reflection as a mediator in this relationship. Indeed, Dimaggio et al. [38] described an association between self-reflection and ToM. The last precondition of insight in our model is stigma-sensitivity. Schizophrenia is associated with a heavy stigma. There is evidence that some patients cope with the threat that stigma poses to their self-esteem by denying the illness [39,40].

Psychological defensiveness in psychosis is associated with unawareness of having a mental disorder, unawareness of the effects of antipsychotic medication and inability to attribute symptoms to a mental disorder [26]. Cooke et al. [23] also reported an association between better insight and lower self-esteem (but not depression) and implied the influence of a psychological mechanism that preserves self-esteem. In addition, unawareness of having a mental disorder is associated with more denial of common personal failings [41]. Apparently, some patients are reluctant, at an unconscious level, to reflect upon themselves in the light of a severe mental illness. In line with our model, patients with lesser abilities for self-reflection and patients who endorse stereotypes about mental illness tell more impoverished stories about themselves [12]. The model is in line with Beck's [6] concept of cognitive insight. Cognitive insight is seen as a prerequisite of insight and encompasses the capacity of patients with psychosis to distance themselves from their psychotic experiences, reflect on them, and respond to corrective feedback. This concept clearly overlaps with the concepts self-reflection, non-social cognition and perspective-taking in our model.

We used this model to develop an intervention to improve insight in schizophrenia. This group-based intervention, from now on referred to as REFLEX, consist of three modules of four sessions each. The central theme of the first module is dealing with stigma. The second and third module aim to stimulate self-reflection through structured exercises. These exercises facilitate mental flexibility and perspective-taking. In the second module, patients reflect upon differences between their past and present circumstances and attributes. In the third module, patients are required to reflect upon their thoughts and feelings in the present. The present paper presents the design of a randomized controlled multicenter trial aiming to evaluate the efficacy of REFLEX.

### Research aims

Main aim of the study is to evaluate the efficacy of REFLEX. Primary outcome measures in this evaluation are the preconditions of insight as specified by our model, while insight is the secondary outcome measure of our trial. Effects of REFLEX on quality of life, self-esteem and mood will be examined as well.

An additional aim of the trial is to examine whether participation in REFLEX will lead to haemodynamic changes, blood oxygenation levels as measured by functional Magnetic Resonance imaging (fMRI), during perspective-taking and self-reflection.

## 2. Methods/Design

The study is funded in part by a European Young Investigator (EURYI) Award from the European Science Foundation to AA. Other contributions (in terms of personnel involved) are from the mental health centers involved. The research has been approved by the medical ethical board of University Medical Center Groningen, Groningen (number: NL2714604209; date: 13-10-2009), and is conducted in accordance with the principles of the Declaration of Helsinki.

### 2.1 Design

The study is a randomized controlled trial, including an intervention group and an active control group. The experimental group consists of patients who participate in REFLEX, the patients in the active control group participate in an adapted form of cognitive remediation for an equal amount of time.

### 2.2 Participants/Setting

A total of 128 patients will be included in the trial. Inclusion criteria for the study are:

• Impaired insight, defined as a) a score of <9 on the Psychosis Inventory (Birchwood et al., 1994) and b) impaired insight rated by a clinician (defined as one or more non-affirmative answers on the following three questions: "Is the patient aware that his/her functioning is suboptimal due to mental illness? Does the patient recognize the symptoms of his condition? Does the patient acknowledge the need for treatment?"). In case of an inconsistency between a) and b) a PANSS interview [5] is administered. Patients with a score > 3 on item G12 pass the threshold for inclusion.

• A diagnosis of schizophrenia according to DSM-IV-TR criteria

• > eighteen years old

• Being able to give informed consent

Exclusion criteria are:

• Receiving CBT at the moment of inclusion

• The presence of a florid psychosis

• A co-morbid neurological disorder

• No competence of the Dutch language

A subsample of 40 patients will also participate in the fMRI part of the study. These patients will have to be eligible for fMRI. Additional exclusion criteria for the fMRI part of the study are: pregnancy or possibility thereof, metal implants in the body, and claustrophobia.

Patients will be recruited in eight mental health institutions in the Netherlands.

### 2.3 Sample size calculation

A previous study on a treatment to improve insight in schizophrenia [42] observed a mean effect size of 0.51 (standardized mean difference). We used this effect size for our power analysis. Sample size was computed using the program developed by David Schoenfeld, Ph.D (Harvard School of Public Health) http://hedwig.mgh.harvard.edu/biostatistics/software.Using the estimated effect size of 0.51, this yielded a total number of 128 patients, with a power of 0.80.

### 2.4 Materials

#### 2.4.1 REFLEX treatment protocol

REFLEX encompasses three modules of four one-hour group sessions each. Module I "Coping with Stigma" focuses on coping with stigmatizing beliefs. The impact of stigmatizing beliefs is discussed and stigmatizing beliefs are disputed and replaced with functional reality-based beliefs about the self. Patients learn that a diagnosis is just a label, saying little about them.

The goal of this module is twofold: first, we presume that denial to cope with the threat that mental illness poses on the self-esteem will be less necessary when the idea of having a mental illness is perceived as less threatening. Following this train of thought, challenging stigmatizing beliefs will ultimately contribute to better insight. Second, with the inclusion of the stigma module we want to prevent an increase of depression to co-occur with increasing insight, as literature has shown that stigma mediates the relationship between insight and mood. In the module "you and your personal narrative" self-reflection is the central theme. Subjects reconstruct their personal narrative, reflect on important changes in their lives and their personal strengths and weaknesses. By offering very structured exercises with clear instructions, REFLEX compensates for cognitive impairments that are thought to hamper self-reflection in schizophrenia. In this module, subjects start practising perspective-taking. Subjects are instructed to ask themselves on a regular basis what other people would think about their thoughts and to check this with an important other. In the third module, called "you in the present", reflection about ongoing thoughts and feelings is stimulated. Between sessions, subjects monitor their thoughts and feelings in their daily life by experience-sampling [43]. In response to a random signal (beeping of a watch) provided six times a day, patients write down the answer to a fixed number of short questions that stimulate self-reflection in a diary. Examples of these questions are: "what was I thinking about before the alarm went off?" and "what would other people think about this thought?". During group sessions, the content of these dairies is discussed. In addition, group exercises and movie vignettes are used to practice perspective-taking during treatment sessions.

#### 2.4.2 Control condition

The control condition of our study consists of twelve group sessions of standardized 'drill and practice' exercises to cognitive functioning. Exercises were adopted from Cognitive Remediation Training protocol [44] that aims to improve cognitive functioning by combining errorless learning (by using tasks varying from extremely easy to easy), immediate feedback, and targeted reinforcement to enhance flexibility, working memory, and planning. Only exercises targeting cognitive functions that are not associated with insight were selected, trainers did not provide feedback on subject's performance.

#### 2.4.3 Screening

*Insight: The Psychosis Insight Scale (PI) *[7]: an eight item self-report questionnaire, consisting of three subscales: awareness of illness; relabeling symptoms to illness, and need for treatment. Total scores range from 0 to 12.

#### 2.4.3 Assessment

##### 2.4.3.1 Behavioral measures

##### Primary outcome measures (preconditions of insight)

As REFLEX aims to improve insight via improving its preconditions, preconditions of insight according to our model are the trials primary outcome measures.

##### Internalized stigma

*The Internalized Stigma of Mental Illness Scale *(ISMI;[45] is a self-rating questionnaire designed to measure the subjective experience of stigma, with subscales measuring Alienation, Stereotype Endorsement, Perceived Discrimination, Social Withdrawal and Stigma Resistance. The ISMI was developed in collaboration with people with mental illnesses and contains 29 Likert items.

##### Self-reflection and mental flexibility

*The Beck Cognitive Insight Scale *(BCIS; [6]) is a self-rating questionnaire developed to evaluate patients' self-reflectiveness and idiosyncratic self-certainty (the ability to consider other possibilities than one's own opinion). The scale consists of 15-items, divided into two subscales: a 9-item self-reflectiveness subscale and a 6-item self-certainty subscale. Total scores are obtained by subtracting the score of the self-certainty subscale from the score on the self-reflectiveness subscale.

##### Self-reflection

*The Self-Reflection and Insight Scale *[46] is a self-rating questionnaire consisting of the factors 'Need for self-reflection', 'Engagement in Self-reflection' and 'Insight'. The scale consists of 20 Likert-scale items.

##### Perspective-taking

The Theory of Mind subscale of the *Davos Assessment of Cognitieve Biases Schaal *(DACOBS) [Van der Gaag, Schütz, Ten Napel, Landa, Delaspaul, Bak & Tsacher, The development of the Daavos Assessment of Cognitive Biases Scale, in preparation] was used to assess perspective-taking. The DACOBS is 42-item Likert self-rating scale that measures cognitive biases and safety behavior in psychosis. It consists of seven subscales: jumping to conclusions, dogmatic bias, selective attention for threat, self-as-target bias, Theory of Mind problems and safety behavior. The Theory of Mind subscale encompasses six items, e.g.: *If I hear other people laugh, I think they are laughing at me*.

#### Secondary outcome measures (Insight)

##### Insight

The *Schedule for Assessment of Insight-Expanded *(SAI-E) [3] an 11-item semi-structured interview to assess insight, based on David's three dimensions of insight. The SAI-E takes both the opinion of the interviewer and the caretakers into account.

##### Insight

Item G12 of *the Positive and Negative Symptom Scale *(PANSS) [5]. Item G12 is one of the thirty items of the PANSS and exist of a seven-point scale, ranging from 1 (very good insight) to seven (no insight). Item G12 of the PANSS is often used to assess insight in psychosis and is highly correlated with other insight measures, such as the SAI, SAI-E and ITAQ [47].

##### Insight

The Beck Cognitive Insight Scale (BCIS, [6]) is a 15-item self-report questionnaire to evaluate patients’ reflectiveness and their overconfidence in their interpretations of their experiences. The 15 items yield a 9-item self-reflectiveness subscale and a 6-item self-certainty subscale. 

#### Other outcome measures (Correlates of insight)

##### Depression

The *Quick Inventory of Depressive Symptomatology Self-Report (QIDS-SR) *is a 16-item self-report questionnaire that rates depressive symptoms according to the DSM-IV in the week before assessment [48].

##### Self-esteem

The *Self-Esteem Rating Scale-Short Form *is a self-report questionnaire that measures self-esteem. It encompasses statements that are linked to social contacts, achievement and competency [49] and is validated for people with schizophrenia.

##### Symptoms

The *Positive and Negative Symptom Scale *(PANSS; [5] was used to measure psychopathology.

##### Quality of Life

The *Self-rating Manchester Short Assessment of Quality of Life *(MANSA; [50] is a 16 Likert-scale item measure derived from the Lancashire Quality of Life Profile [51]. The MANSA consists of four objective questions and twelve subjective questions. The subjective items assess satisfaction with life as a whole, job, financial situation, number and quality of friendships, leisure activities, accommodation, personal safety, people that the individual lives with (or living alone), sex life, relationship with family, physical health and mental health.

##### 2.4.3.2 fMRI

##### Self-reflection

During the self-reflection task subjects view 180 different short sentences (white letters on a black screen), subdivided into three main conditions (60 sentences per condition). Patients are presented statements, which refer to themselves ("self-condition"), to a significant other ("other-condition"), and to semantic knowledge ("baseline condition"). The self-condition is subdivided into four conditions (15 sentences per condition): a 'negative' mental condition (for example sentences as 'I am insensible', 'I forget important things'), a 'positive' mental condition ('I am intelligent', 'I am honest'.), a negative physical condition ('I am often ill', 'I am fat'), and a positive physical condition ('I am strong', 'I am healthy).

The other-condition also includes 'negative' and 'positive' sentences concerning mental qualities or physical qualities. Examples of sentences included in the 'semantic knowledge condition' are 'Milk is red', 'Dogs run faster than snails' and 'Birds eat cats'. The amount of true/false items in this condition is balanced.

##### Perspective-taking

The perspective-taking paradigm is adapted from a paradigm developed by Hooker and colleagues [52]. The paradigm consists of three conditions: a control condition, emotion recognition, and emotion inference. To familiarize patients with the task five practice items for each condition are presented before patients enter the scanner. Each condition consists of 25 images of social scenarios. During the control condition, patients simply have to count the number of people in the scene. For the Emotion Recognition task, patients are required to identify the emotion of a character in the scene that was indicated with a fixation cross. Answers are presented in a four-option multiple choice format. In the Emotion Inference task, patients are asked what the character indicated by the fixation cross would feel if she/he had full knowledge about what is happening in the scene. Half of the characters in this condition holds a false belief. Answers are once more presented in a four-option multiple choice format. In both the Emotion Recognition and the Emotion Inference task emotional valence is balanced within the emotion recognition and emotion interference condition. Two parallel versions of the paradigm were developed, to prevent practice effects and for pre- and post treatment testing

### 2.5 Procedure

Patients who fulfill the inclusion criteria, will be invited to participate in the study. If a patient is willing to participate, study procedures will be explained in detail and after a period of two weeks written informed consent is obtained. Subsequently, diagnosis is verified by the Mini Plus, a semi-structured interview to assess DSM IV pathology [53]. Thereafter, patients are randomly allocated to REFLEX or control condition. Randomization procedures start when the required number of patients per center (ranging from 17-19) are included, or when the first patients was included more than six weeks ago while >10 people are included.

Randomization is centrally coordinated by the Trial Coordination Center of the University Medical Hospital Groningen. The project coordinator gives the subject a unique code and these codes are entered for each patient into a computerized systematic program by an independent researcher. Results of the randomization process are passed to the project coordinator in sealed envelopes and distributed to the on-site therapists. Subsequent subjects are randomized in blocks of two or four, to ensure that the number of patients will be balanced over conditions. Assessment takes place before (T1), directly after (T2) and six months after the training (T3). Assessors are not aware of the condition (control or treatment) the subject is in. During the entire trial patients receive treatment as usual, with the exception of cognitive behavioral therapy.

All fMRI scans will be made in the Neuroimaging Center of the UMCG in Groningen, right before (T1) and directly after (T2) treatment. For geographical reasons, recruitment for the fMRI study is limited to the institutions located in the North of the Netherlands: GGZ Drenthe, Assen; UMCG and Lentis, Groningen, and GGZ Friesland, Leeuwarden. Patients who participate in the fMRI study will be randomized separately.

## 3. Statistical analysis

### 3.1 Behavioral data

Analysis will be performed according to the *intention to treat principle*. Differences in scores on each of the dependent variables will be examined for T1-T3. The significance of possible differences will be tested with logistic multilevel modeling [54] with the condition (REFLEX or Control) and treatment phase (T1-T3) as levels. A model will be built for each of the dependent variables. Dummy variables will be created for each level and the statistical significance of the regression effects will be tested using the approximate t-test. The dummy variables and their interaction are entered as fixed effects in the model. As random effects, the between-individual and within-individual variance were estimated. All models will be built using the program MlwiN.

### 3.2 fMRI

Scans will be acquired using a 3T Phillips Intera Quaser (Best, The Netherlands) equipped with a synergy SENSE eight-channel head coil. Functional images are acquired using a T2*-weighted echo-planar sequence with 37 interleaved axial slices oriented approximately 10-20° to the ac-pc transverse plane, a thickness of 3.5 mm and no slice gap to cover the entire cortex (TR = 2 s, TE = 35 ms, flip angle = 70 degrees, FOV = 224 mm, 64 × 64 matrix of 3.5 × 3.5 × 3.5 voxels). In addition, two T1-weighted 3-D fast field echo (FFE) anatomical images (voxel size, 1 × 1 × 1 mm) containing 160 slices (TR = 25 ms; TE = 4.6 ms; slice-thickness = 1 mm; 256 × 256 matrix; FOV 26 cm) will be acquired parallel to the bicommissural plane. Data will be preprocessed using the Statistical Parametric Mapping software package (SPM8, Wellcome Department of Cognitive Neurology, London, UK: http://www.fil.ion.ucl.ac.uk) in the following order: functional images will be corrected for slice timing, realigned to the first volume of the first run to correct for shifts in head position and coregistered to the anatomy. Coregistrations were controlled manually for each subject to ensure correct coregistration. Functional images were spatially normalized based on the basis of the MNI (Montreal Neurological Institute) T1 template and then spatially smoothed with a 10 mm full-width half-maximum (FWHM) isotropic Gaussian Kernel. Preprocessed data will be analyzed to calculate the main effects of Condition and the two-way interaction of Condition x Phase.

## 4. Discussion

REFLEX may have a potential for improving insight in patients with schizophrenia. Our design offers the opportunity not only to examine the results of REFLEX at a behavioral level, but takes underlying changes in brain activation into account as well. With the study, we hope to contribute to the existing knowledge of what mechanisms are underlying changes in insight in schizophrenia. Improving insight in schizophrenia is a challenging task that needs careful consideration. During the development of the intervention, some clinicians addressed that patients might become more depressed if their insight would improve. Although the evidence for the development of depression is not conclusive [23], we paid special attention to this issue. As was explained in the introduction of this paper, there is evidence that this risk concerns patients with internalized stigma. By including a module that aims to reduce internalized stigma, we feel we have minimized this potential risk. Second, by definition, patients with impaired insight often do not feel they need treatment and will not spontaneously enroll in a therapy trial to improve their insight. Therefore, a common language needed to be developed. We cannot simply give patients a phone call, tell them that they are mentally ill and not fully aware of this and expect them to participate in our study. Instead, patients will be explained that being under treatment in a mental health institution brings about a lot of changes in their daily lives. REFLEX may help them to recognize these changes and gain more control over their lives. In a previous and unpublished pilot study we found that explaining the aim of REFLEX in comparable phrasing was acceptable to most patients and made them consider participation. Third, a methodological problem that is associated with insight is that most assessment instruments are based upon the traditional medical model: patients have to use the same terminology as their psychiatrists to be considered insightful. This approach ignores the insight some patients demonstrate, when they are able to describe the problems they experience in daily life and to attribute these problems to a mental illness. If they do not use the term "psychosis" or "schizophrenia" to describe their mental health, their insight is rated as impaired by most of the current instruments. However, this may be just a discussion about labels and not about actual insight. We hope to have solved this problem by including scales that measure the preconditions of insight, such as the BCIS that measures cognitive insight, to traditional insights scales. The BCIS does not measure agreement insight in and medical way, but takes into account how patients perceive their own thoughts and feelings. Finally, care as usual for people with schizophrenia in the Netherlands is extensive. Psycho-education, Liberman training modules (social skills), and CBT are accessible for most patients. Because of this extensive care, it is very hard to obtain treatment effects over and above the effect of care that is already provided. However, several studies have shown that specific interventions can make a significant contribution to relevant outcome measures [55,56]. Through the unique focus in REFLEX on the improvement of self-reflectivity and perspective taking abilities and by that improving insight we expect to make a significant contribution to the well-being of people with schizophrenia.

## Competing interests

The authors declare that they have no competing interests.

## Authors' contributions

MP and AA conceived the study and designed the study with advice from MG and CB. MP wrote the manuscript and is the study's principal investigator. MP developed the REFLEX treatment protocol with significant contributions from AA, MG and CB. LM is involved the fMRI part of the study, that is supervised by AA. All authors read and approved the final manuscript.

## Pre-publication history

The pre-publication history for this paper can be accessed here:

http://www.biomedcentral.com/1471-244X/11/161/prepub
